# Hospital Support: Individual and Collective Efforts

**Published:** 1902-11-22

**Authors:** 


### The Hospital.

A Journal of

The Medical Sciences and Hospital Administration.

Vol. XXXIII.—No. 843.] EDITED BY SIR HENRY BURDETT, K.C.B., AND SOLOMON C. SMITH, M.D., M.R.C.P. [November 22, 1902.

### HOSPITAL SUPPORT: INDIVIDUAL AND COLLECTIVE EFFORTS.

The steady progress and commanding success financially of the King's Hospital Fund has caused some people to believe that King Edward's Fund will gradually attract the great bulk of the financial support given by the public to hospital work. In other words, the contention now is that individual effort, as represented by the managers of each separate hospital, will be rendered largely ineffective by the success achieved by the collective efforts of those responsible for the management of this great central fund. It would be an evil day for the voluntary hospitals of London and a calamity for the King's Fund, were it not certain that the near future will prove the critics to be as wrong in the views they now propound, as they have been proved wrong in regard to those they originally promulgated on the institution of the Fund in the Diamond Jubilee Year. It may be worth while to recall the purpose for which the King's Fund was originally instituted, and to consider why these contentions are never likely to be fulfilled.

The King's Hospital Fund was instituted by a letter dated February 5th, 1897, issued by King Edward VII., then Prince of Wales, and signed by him. That letter sets forth that the finances of the hospitals of London have long been a source of anxiety and solicitude, that the deficiency in the ordinary receipts compared with the ordinary expenditure of 122 metropolitan hospitals and convalescent homes, when the figures were limited to institutions which failed to meet their outgoings, amounted to upwards of £100,000. Whilst not trenching upon the ground occupied by the Hospital Sunday and Saturday Funds, the efforts of the Council of the King's Hospital Fund were to be concentrated upon an endeavour to secure from £100,000 to £150,000 in annual subscriptions from those who had not hitherto regularly contributed anything to the hospitals. Subscriptions were consequently invited of 1s. per annum and upwards from all these classes, and the King expressed his confidence that the combined appeal on behalf of the hospitals of London, setting forth their work in its magnitude and importance, would prove irresistible.

It will be noticed that the King's Fund was instituted to supplement the contributions already received by the hospitals by tapping a new source of revenue, and so widening the area of givers. As the King stated in his letter at the time his Fund was founded, the contributors to all the London hospitals numbered less than one in a hundred of the population. There was therefore an abundant field for the King's Fund to cultivate, and it is admitted by those who know the facts most accurately, that the King's Fund has been so carefully administered, that no single hospital has suffered any appreciable diminution of its income from voluntary sources, whilst those London hospitals which are the most ably administered are, in fact, able to point to an increase in the voluntary offerings which they have received during the last four years. Nor has the Metropolitan Hospital Sunday Fund suffered, for the amount received each year has been above the average, and during the present year the sum collected was a record one, not only in amount, but from the circumstance that the receipts from the congregations were £10,000 more than in either of the previous two years, and £6,000 more than had ever been received before from the congregations during the thirty years of its existence.

It is therefore clear, despite its gratifying success, that King Edward's Fund has not attracted the great bulk of the financial support given by the public to hospital work. On the contrary, it has loyally fulfilled the purposes for which it was established, and, by increasing the number of givers and awakening an interest in hospitals amongst the masses of the people who had heretofore taken little or no interest in their work, it has immeasurably strengthened the voluntary principle of hospital support, and so conferred a lasting benefit upon every London hospital. Again, the amount which the King's Hospital Fund was established to raise, *i.e.*, a maximum of £150,000 a year, only represented the carefully calculated deficiency which had to be supplied in order to place each of the London hospitals upon a sound financial basis. Already nearly two-thirds of this deficiency is secured to the hospitals by the King's Fund, and there is the hope that through the League of Mercy the whole £150,000 a year will be secured within the next five years.

When this result has been achieved the conductors of the King's Fund will merely have to secure the continued confidence of the public to maintain the Fund at this amount, and each hospital, with the aid of the grants it receives from the central funds, will be placed in a position to maintain itself successfully by the voluntary contributions raised by its own efforts. It would indeed be a calamity were it possible to conceive the time when the existence of any central fund, and its success should tend to destroy the individual as opposed to the collective efforts of hospitals, because these individual efforts must prove immensely beneficial to the hospitals themselves as well as to the public who depend upon them for medical aid. The reason why the general hospitals in England are rightly regarded with admiration all the world over, is because the voluntary principle has secured, better than any other system, the continuous development of everything which tends to make a hospital efficient, progressive in regard to matters of treatment, and successful in every respect from the patients' point of view. In such circumstances it is essential that the public should clearly understand that the success of the King's Fund means adequate success too for each London hospital, by rendering the individual efforts of its managers so successful as to make its financial basis secure for all time.

